# A randomised trial of intrapericardial bleomycin for malignant pericardial effusion with lung cancer (JCOG9811)

**DOI:** 10.1038/sj.bjc.6604866

**Published:** 2009-01-20

**Authors:** H Kunitoh, T Tamura, T Shibata, M Imai, Y Nishiwaki, M Nishio, A Yokoyama, K Watanabe, K Noda, N Saijo, JCOG Lung Cancer Study Group, Tokyo, Japan

**Affiliations:** 1 Department of Medical Oncology, National Cancer Center Hospital, 5-1-1 Tsukiji, Chuo-ku, Tokyo 104-0045, Japan; 2 JCOG Data Center, Center for Cancer Control and Information Services, National Cancer Center, 5-1-1 Tsukiji, Chuo-ku, Tokyo 104-0045, Japan; 3 Department of Thoracic Oncology, National Cancer Center Hospital East, 6-5-1 Kashiwanohara, Kashiwashi, Chiba 277-8577, Japan; 4 Department of Medical Oncology, Cancer Institute Hospital, 3-10-6 Ariake, Koto-ku, Tokyo 135-8550, Japan; 5 Department of Medical Oncology, Niigata Cancer Center, 2-15-3, Kawagishi-cho, Niigata-shi, Niigata 951-8566, Japan; 6 Department of Respiratory Medicine, Yokohama Municipal Citizen’s Hospital, 56 Okazawa-cho, Hodogaya-ku, Yokohama, Kanagawa 240-8555, Japan; 7 Division of Thoracic Oncology, Kanagawa Cancer Center, 1-1-2 Nakao, Asahi-ku, Yokohama, Kanagawa 241-0815, Japan; 8 National Cancer Center Hospital East, 6-5-1 Kashiwanohara, Kashiwashi, Chiba 277-8577, Japan

**Keywords:** malignant pericardial effusion, lung cancer, drainage, sclerosis, intrapericardial instillation, bleomycin

## Abstract

Safety and efficacy of intrapericardial (ipc) instillation of bleomycin (BLM) following pericardial drainage in patients with malignant pericardial effusion (MPE) remain unclear. Patients with pathologically documented lung cancer, who had undergone pericardial drainage for MPE within 72 h of enrolment, were randomised to either arm A (observation alone after drainage) or arm B (ipc BLM at 15 mg, followed by additional ipc BLM 10 mg every 48 h). The drainage tube was removed when daily drainage was 20 ml or less. The primary end point was survival with MPE control (effusion failure-free survival, EFFS) at 2 months. Eighty patients were enrolled, and 79 were eligible. Effusion failure-free survival at 2 months was 29% in arm A and 46% in arm B (one-sided *P*=0.086 by Fisher’s exact test). Arm B tended to favour EFFS, with a hazard ratio of 0.64 (95% confidence interval: 0.40–1.03, one-sided *P*=0.030 by log-rank test). No significant differences in the acute toxicities or complications were observed. The median survival was 79 days and 119 days in arm A and arm B, respectively. This medium-sized trial failed to show statistical significance in the primary end point. Although ipc BLM appeared safe and effective in the management of MPE, the therapeutic advantage seems modest.

Malignant pericardial effusion (MPE) is a grave complication of malignant tumours. The frequency of pericardial involvement by malignancy has been estimated to be 10–21% at autopsy (Theologides, 1978; Klatt and Heitz, 1990).

Malignant pericardial effusions are often asymptomatic and detected incidentally by echocardiography or computed tomography. Symptomatic cases, however, often manifest cardiac tamponade, which can rapidly lead to cardiovascular collapse and death, unless promptly treated (Press and Livingston, 1987).

Lung cancer is the most frequent cause of MPE, and other common primary sites include breast cancer, oesophageal cancer, lymphoma and leukaemia (Abraham *et al*, 1990; Wilkes *et al*, 1995; Yonemori *et al*, 2007). The prognosis of MPE in lung cancer patients is particularly poor, with a reported median survival of 3 months or less (Okamoto *et al*, 1993; Gornik *et al*, 2005).

Although prompt diagnosis and pericardial drainage result in good palliation of symptoms, drainage alone is often inadequate to prevent re-accumulation of the fluid after the drainage tube is removed (Shepherd, 1997). There are numerous reports of pericardial sclerosis for MPE by the instillation of various agents, such as tetracycline/doxycycline (Shepherd *et al*, 1987; Maher *et al*, 1996), a streptococcal preparation (Imamura *et al*, 1991), bleomycin (BLM) (Vaitkus *et al*, 1994; Liu *et al*, 1996; Maruyama *et al*, 2007), thiotepa (Colleoni *et al*, 1998; Martinoni *et al*, 2004), cisplatin/carboplatin (Moriya *et al*, 2000; Tomkowski *et al*, 2004), 5-fluorouracil (Lerner-Tung *et al*, 1997), anthracyclines (Kawashima *et al*, 1999), vinblastine (Primrose *et al*, 1983), mitoxyantrone (Norum *et al*, 1998), mitomycin C (Kaira *et al*, 2005) and ^32^P-colloid (Dempke and Firusian, 1999), after drainage. Platinum agents are actually not ‘classic’ sclerosants to induce inflammatory adhesion of the pericardial sac; they were apparently used as local chemotherapy. Whereas each study reports favourable outcomes in terms of MPE control and prevention of re-accumulation, almost all were performed as phase II trials, and no definite conclusions could be drawn (Press and Livingston, 1987; Vaitkus *et al*, 1994).

In one of the very few randomised trials conducted to date, Liu *et al* (1996) reported that BLM is the preferred agent for sclerosis, because of the lower morbidity associated with it. However, to the best of our knowledge, the efficacy and safety of pericardial sclerosis itself has never been evaluated by a prospective randomised trial.

This trial was aimed at evaluating the safety and efficacy of pericardial sclerosis induced by intrapericardial (ipc) BLM instillation, as compared with pericardial drainage alone, in lung cancer patients with MPE.

## Patients and methods

### Patient eligibility criteria

Patients with pathologically documented lung cancer, who had undergone pericardial drainage for clinical MPE (moderate to large accumulation of fluid), were eligible for study entry. Indications for the drainage were clinically determined; cases after emergent drainage and those after elective one were both included. Patient registration should be done within 72 h of drainage. The eligibility criteria were as follows: 75 years of age or less, expected life prognosis of 6 weeks or more with control of the MPE and minimum organ functions (leukocyte count⩾3000 per mm^3^, platelet count⩾75 000 per mm^3^, haemoglobin⩾9.0 g dl^−1^ and no renal or hepatic failure; however, laboratory abnormalities related to cardiac tamponade were allowed). Patients with chemotherapy-naive small cell cancer were excluded. Other exclusion criteria included apparently non-malignant effusion (e.g., purulent effusion), recurrent MPE, myocardial infarction or unstable angina within the previous 3 months, constrictive pericarditis, active interstitial pneumonia, severe infection and disseminated intravascular coagulation. Those with an unstable clinical condition attributable to other severe complications, such as superior vena cava syndrome, central airway obstruction or uncontrollable massive pleural effusion, were also excluded.

Patient eligibility was confirmed by the Japan Clinical Oncology Group Data Center before patient registration. The study protocol was approved by the institutional review boards at each participating centre and all the patients provided written informed consent.

### Treatment plan

The study protocol did not limit the method used for the pericardial drainage. Both percutaneous tube pericardiostomy (non-surgical method), in which a drainage catheter is inserted using the Seldinger technique, and subxiphoid pericardiostomy (surgical method), in which a drainage tube is placed surgically, were allowed; each participating institution, however, basically adhered to one method, which they used in routine practice. The drainage method used was recorded on the case report form.

After registration with telephone or facsimile, the patients were randomly assigned to one of the two treatment arms with block randomisation stratified by the institution. In arm A, no additional intervention was performed and the patient was observed clinically after the pericardial drainage. In arm B, 15 mg of BLM dissolved in 20 ml of normal saline was instilled through the drainage catheter into the pericardial space immediately after the patient registration. The catheter was then clamped and reopened after 2 h, allowing resumption of the drainage. Additional doses of BLM at 10 mg were instilled similarly every 48 h, unless the criteria for tube removal, as described below, were met.

The drainage tube was removed, in both arm A and arm B, when the drainage volume per 24 h was 20 ml or less. If the criterion was met during the 24 h preceding randomisation in a patient allocated to arm A, the tube was immediately removed.

### Patient evaluation and follow-up

Primary control of the MPE was considered to be achieved when the drainage tube could be successfully removed within 7 days of randomisation. When the criterion for tube removal, that is 20 ml per 24 h, could not be met by 7 days, the case was judged to show primary failure of the protocol therapy: treatment after off-protocol was not limited by the study protocol. When the drainage tube had to be removed because of obstruction, but re-drainage was clinically unnecessary, it was judged to have been successfully removed with primary control of MPE.

Monitoring for recurrence of the MPE in those who showed primary control was conducted by echocardiography at 1, 2, 4, 6 and 12 months. When the estimated fluid volume in the recurrent effusion exceeded 100 ml, the case was labelled as showing MPE re-accumulation and recurrence. Re-drainage was performed as clinically indicated.

The adverse effects of the therapy were evaluated according to the Japan Clinical Oncology Group Toxicity Criteria (Tobinai *et al*, 1993), modified from the National Cancer Institute Common Toxicity Criteria version 1.

The primary end point of the study was effusion failure-free survival (EFFS) rate at 2 months; EFFS was patient survival without MPE recurrence as defined above, in patients showing primary control. It was calculated as the period from the date of pericardial drainage to the date of MPE recurrence or the patient’s death. For those patients with primary failure, MPE recurrence was considered to have occurred at the date of drainage, with an EFFS of zero. Effusion failure-free survival was judged regardless of the other disease status.

The secondary end points included the primary MPE control rate, time to drainage tube removal, EFFS, treatment-related morbidity, proportion of late pericardial or cardiac complication, overall survival (OS) and symptom scores.

Study-specific four-item symptom scores were completed by patients at the time of randomisation (i.e., after pericardial drainage) and at 1 month after the enrolment. The scores were to be interviewed by the health professionals other than the attending physicians. The items consisted of cough, pain, anorexia and shortness of breath. The scoring was conducted as follows: as not at all present (0), a little (1), moderate (2) and very much (3). The score for each item and the sum of the total score for all the four items were compared between the baseline and the follow-up assessments, and judged to be improved (lower scores in the follow-up assessments), stable (no change of scores) or worsened (higher scores, or the patient could not fill out the questionnaire, in the follow-up assessments).

### Statistical considerations

From the historical data, the EFFS rate at 2 months in arm A was assumed to be 30% and that in arm B was presumed to be 60%. The study was designed to provide 80% power with 5% one-sided *α*. The required sample size was calculated as 80 patients, 40 in each arm, for comparing independent proportions.

The OS, time to tube removal and EFFS of both arms were calculated by the Kaplan–Meier method and compared by log-rank tests. The primary MPE control rate, symptom scores, complication rates and EFFS at each of the follow-up points were compared using Fisher’s exact test. All analyses were performed with the SAS software version 9.1 (SAS Institute, Cary, NC, USA).

## Results

### Patient characteristics and treatment delivery

From August 1999 to January 2006, 80 patients from 14 institutions were enrolled and randomised, 42 to arm A and 38 to arm B. One patient in arm B was found to be ineligible because of late registry, 2 weeks after the pericardial drainage. All 80 patients were analysed for their characteristics and chemotherapy morbidity, and the 79 eligible patients were analysed for efficacy and survival.

Table 1 lists the characteristics of the patients, which were generally well balanced between the arms, except for the effusion cytology: there were numerically more patients with cytology-positive effusions in arm A. Cytology of the effusion was positive in 58 cases out of the 76 examined (76%).

In arm B, all 38 patients received at least one ipc BLM instillation and a total of 74 administrations: seven patients received four administrations (total BLM dose: 45 mg), five received three administrations (total BLM: 35 mg), five received two administrations (total BLM: 25 mg) and the remaining 21 received a single administration (total BLM: 15 mg). There was no apparent relationship between total dose and efficacy end points such as EFFS, except that those required four administrations had a worse primary control of the MPE.

A total of 24 patients (14 in arm A and 10 in arm B) received systemic chemotherapy after drainage tube removal. Nine patients (five in arm A and four in arm B) received gefitinib. Cytotoxic chemotherapy was administered to 21 patients (11 in arm A and 10 in arm B).

### Morbidity and early deaths

Table 2 summarises the morbidity of the protocol therapy. Although 30 (38%) of the patients experienced some pain, no significant difference in the incidence and severity of pain was observed between the arms. Bleeding and infections were rare and generally controllable. Two patients in arm B developed transient fever of moderate degree (38–38.7°C). One case with constrictive pericarditis at 4 months and another with late cardiac dysfunction at 12 months after the registry, both reported to be grade 2, were observed in arm B.

As anticipated, there were as many as nine early deaths within 30 days of randomisation; five in arm A and four in arm B. Although the death was ascribed to disease progression in the majority, two patients in arm A died of massive bleeding during surgical attempts at re-drainage for recurrent MPE, possibly due to crack formation in the ventricular wall upon dissection of the adherent pericardium. Another patient in arm B died suddenly on day 12 of the protocol without a clear cause.

### Efficacy end points

**Figure 1:**
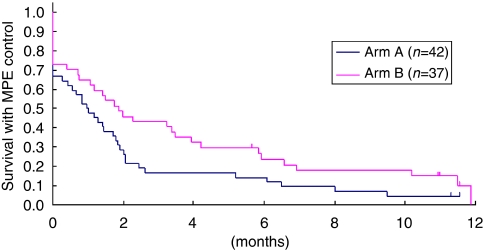
Effusion failure-free survival (EFFS). The median EFFS was 30 days in arm A and 57 days in arm B, with a hazard ratio of 0.64 (95% confidence interval: 0.40–1.03), with arm B significantly favouring this parameter (one-sided *P*=0.030 by log-rank test).

Primary control of the MPE with successful tube removal within 7 days of randomisation was achieved in 28 of the 42 cases (67%) in arm A and 27 of the 37 eligible cases (73%) in arm B, the difference between the two groups not being statistically significant. The median time to tube removal was 7 days in each arm. Arm B favoured EFFS (Figure 1), with a hazard ratio of 0.64 (95% confidence interval: 0.40–1.03, and one-sided *P*=0.030 by log-rank test).

The EFFS at 1, 2, 4, 6 and 12 months was 50, 29, 17, 14 and 5%, respectively, for arm A, and 65, 46, 32, 24 and 10%, respectively, for arm B. Although arm B also favoured the primary end point, EFFS at 2 months (46 *vs* 29%), the difference between the two groups was not statistically significant (one-sided *P*=0.086 by Fisher’s exact test).

The median OS was not significantly different between the two arms: 79 days in arm A and 119 days in arm B. The OS rates at 6 months were 27 and 31% in arm A and arm B, respectively.

### Subgroup analysis

**Figure 2:**
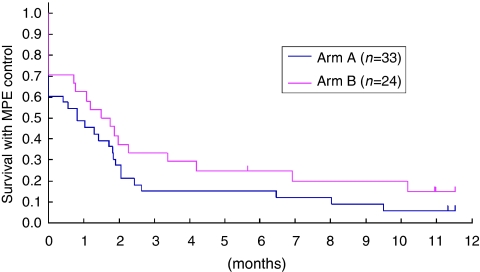
Effusion failure-free survival (EFFS) in effusion cytology-positive patients. In the effusion cytology-positive patient subset, arm B favoured EFFS. The hazard ratio was 0.69 (95% confidence interval: 0.39–1.21).

**Figure 3:**
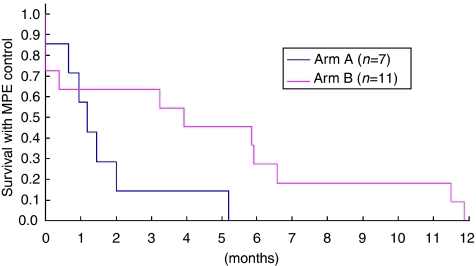
Effusion failure-free survival (EFFS) in effusion cytology-negative or -indeterminate patients. In the effusion cytology-negative or -indeterminate patient subset, arm B favoured EFFS. The hazard ratio was 0.39 (95% confidence interval: 0.12–1.21).

As more patients in arm A had cytology-positive effusion, which has been reported to be associated with a poor prognosis (Gornik *et al*, 2005), subset analysis was performed according to the effusion cytology status (Figures 2 and 3). In both cytology-positive patients (Figure 2) and cytology-negative or -indeterminate patients (Figure 3), arm B favoured EFFS.

**Figure 4:**
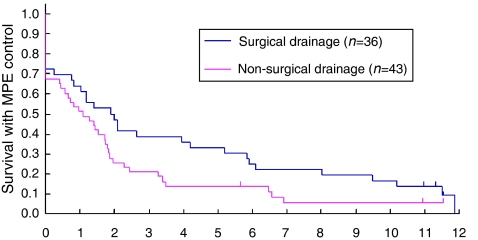
Effusion failure-free survival (EFFS) and drainage method. Patients with surgical drainage tended to have longer EFFS (median EFFS: 2.0 *vs* 1.1 month).

Thirty-six patients had undergone surgical (subxiphoid pericardiostomy) and 43 had undergone non-surgical (percutaneous tube pericardiostomy) drainage before randomisation. Patients with surgical drainage tended to have a longer EFFS (Figure 4). The effect of ipc BLM was observed irrespective of the drainage method employed; arm B tended to favour EFFS both in patients with surgical drainage (hazard ratio 0.62, 95% confidence interval: 0.30–1.29) and in those with non-surgical drainage (hazard ratio 0.56, 95% confidence interval: 0.29–1.05).

### Symptom palliation

The baseline symptom scores were taken for all of the 79 eligible patients, at enrolment (after drainage). At the 1-month follow-up, approximately half of the patients (55% in arm A and 51% in arm B) had stable or improved overall scores. There were no significant differences between the arms for any of the symptom scores (Table 3).

## Discussion

Malignant pericardial effusion is a potentially life-threatening complication of malignancy that usually manifests itself at an advanced or terminal stage of the disease. It brings great agony to the patient once it becomes symptomatic, with dyspnoea, orthopnoea, chest pain and cough. Although the prognosis of the patients with MPE is very poor, especially in those with chemotherapy-resistant tumours such as non-small-cell lung cancer (Press and Livingston, 1987; Okamoto *et al*, 1993; Gornik *et al*, 2005; Yonemori *et al*, 2007), optimal management is very important for palliation.

Pericardial sclerosis following drainage has been widely performed. However, data are available mainly from phase II trials or case series. In fact, historical comparison has failed to demonstrate the efficacy of pericardial sclerosis over drainage alone (Okamoto *et al*, 1993; Vaitkus *et al*, 1994). It has also been suggested that sclerosis may be effective in preventing re-accumulation of MPE after percutaneous tube pericardiostomy, but not after subxiphoid pericardiotomy, because the surgical intervention alone was considered to be sufficient to prevent recurrent MPE (Press and Livingston, 1987; Park *et al*, 1991; McDonald *et al*, 2003).

In addition, there are some potential morbidities associated with pericardial sclerosis; most of the agents used as sclerosants produce unpleasant adverse effects, such as fever and pain (Liu *et al*, 1996). There is also concern about the complications of the procedure, both in the short term, such as bleeding and infection, and in the long term, such as constrictive pericarditis, as the inflammatory response causes adhesion of the visceral and parietal pericardium (Shepherd, 1997).

We undertook a randomised trial to evaluate the efficacy of pericardial sclerosis following drainage as compared with drainage alone. We chose BLM as the sclerosant agent for ipc instillation, because of its low toxicity as compared with doxycycline, reported from an earlier randomised trial (Liu *et al*, 1996). We included only patients with non-small-cell lung cancer or chemotherapy-treated small cell cancer to minimise the influence of systemic chemotherapy after the protocol study (Vaitkus *et al*, 1994). We randomised the patients after the pericardial drainage, as we judged that obtaining informed consent before it, that is when the patients suffer from symptoms of MPE, would be very difficult. Therefore, we did not specify the indication for drainage and enrolled cases after both emergent and elective drainage. We thus focused on the prevention of MPE recurrence. We could not find any comparable phase III trial on this participant, and no such trial is registered in ClinicalTrial gov.

We found that ipc BLM instillation seemed to be effective at preventing the recurrence of MPE. However, the benefit in the primary end point, that is, EFFS at 2 months, was not significantly different, which is a major drawback to make a definitive conclusion. The therapeutic benefit, which could not be demonstrated with our modestly sample-sized trial, therefore, might be only a modest one. On the other hand, the benefit of ipc BLM seemed to be unrelated to the drainage method. As expected, the OS was poor in both arms and not significantly different.

Our study has several limitations. One is that without significant survival prolongation and difference of symptom scores, modest improvement of the EFFS might not represent true patient benefit. We believe, however, that conductance of our trial itself would be fully justified; given the severe symptoms of uncontrolled MPE and the inconvenience of the drainage tube, survival without MPE would be a worthwhile treatment goal.

The second limitation was that we limited the participants to lung cancer patients, which makes it difficult to evaluate late complications due to short OS. In patients with more chemotherapy-sensitive tumours such as breast cancer or lymphoma, many more patients may be expected to live for up to at least 1 year longer. There would be greater concern about late pericardial or cardiac complications, which we did observe in two of our own cases. Even for lung cancer patients, advances in systemic therapy may be expected to improve the outcome of those with even far-advanced disease in the future, which would evidently modify the risk/benefit of ipc BLM.

The third limitation of our study was that we did not control for the method of primary pericardial drainage, and each institution chose it in accordance with its daily practice. We do not believe that our results were much biased by the drainage methods, as each participating institution basically adhered to one method of its choice, and the ipc BLM arm tended to favour EFFS in both subgroups with surgical and non-surgical drainage. However, control for the drainage method or indication (emergent *vs* elective) for drainage might be necessary in future trials, as they might well affect the patient outcomes. In fact, we did observe that, although not a randomised comparison and thus it should be interpreted with caution, patients who underwent surgical drainage tended to have a better MPE control.

Recently, less invasive techniques for surgical treatment of MPE have been described, such as percutaneous balloon pericardiotomy (Ziskind *et al*, 1993; Wang *et al*, 2002), which create a pleuro-pericardial communication and allow fluid drainage into pleural space. It was reported to be effective and safe, and may potentially obviate the need for surgical intervention. However, it has yet to be compared with other drainage methods and its role has not been established. No patient underwent this procedure in our study.

One ancillary finding of our study was that two patients died of major bleeding during surgical attempts at re-drainage for recurrent MPE. Although it has rarely been reported in the literature, partial adhesions could have led to injury to the cardiac wall during the surgical procedure.

In this trial, we evaluated the safety and efficacy of pericardial sclerosis with a ‘classic’ sclerosant agent of BLM. Future trial designs would include one to compare BLM with another agent with a different mode of action, such as intrapericardial instillation of a platinum compound as ‘local chemotherapy’.

In conclusion, we found that pericardial sclerosis with ipc BLM after drainage appears to be safe and effective, overall, in the management of MPE in patients with lung cancer and should be a valid therapeutic option in these patients. We could not, however, demonstrate a statistical significance in the primary end point with the modest sample size of 80. The therapeutic advantage might not be large enough, and more trials are warranted.

## Conflict of interest

The authors have no conflicts of interest to declare.

Registered in www.clinicaltrials.gov, ClinicalTrials.gov number, NCT00132613 and in UMIN-CTR[www.umin.ac.jp/ctr/], identification number, C000000030.

## Figures and Tables

**Table 1:** Patient characteristics

**Arm**	**A (drainage alone)**	**B (ipc BLM)**
*N*	42	38
		
*Gender*		
Male	27	24
Female	15	14
		
Median age (range)	60.5 (39–75)	60 (42–73)
		
*Histology*		
Small cell	3	2
Non-small cell	39	36
		
*Prior chemotherapy*		
Yes	29	24
No	13	14
		
*Prior thoracic radiotherapy*		
Yes	11	9
No	31	29
		
*Drainage methods*		
Surgical	19	17
Others	23	21
		
Median drainage volume in ml (range)	550 (250–1750)	600 (130–1930)
		
*Effusion cytology*		
Negative	6	11
Indeterminate	1	0
Positive	33	25
Not examined	2	2

ipc BLM=intrapericardial bleomycin instillation.

**Table 2:** Morbidity of the protocol therapy

**Arm**	**A (drainage alone)**	**B (ipc BLM)**
*N*	42	38
		
*Pain*		
None	25	25
Medication not required	4	4
Controlled with non-opioid analgesics	9	7
		
Controlled with opioid analgesics	4	2
		
Uncontrollable	0	0
		
*Infection*		
None	39	35
Controllable	3	3
Uncontrollable	0	0
		
*Bleeding*		
None	42	36
Controllable	0	1
Severe	0	1
		
*Late complications*		
None	42	36
Pulmonary	0	0
Cardiac function	0	1 (grade 2)
Constrictive pericarditis	0	1 (grade 2)

ipc BLM=intrapericardial bleomycin instillation.

**Table 3:** Symptom palliation

**Arm**	**A (drainage alone)**	**B (ipc BLM)**
*N* eligible	42	37
		
*% of those with improved or stable scores* a		
Cough	60%	57%
Pain	50%	62%
Anorexia	55%	62%
Dyspnoea	62%	46%
Total	55%	51%

ipc BLM=intrapericardial bleomycin instillation.

a The scores at 1 month were compared with those at enrolment.

## Appendix

Supported by the Grant-in-Aid for Cancer Research from the Ministry of Health, Labour and Welfare of Japan (11S-2, 11S-4, 14S-2, 14S-4, 17S-2, 17S-5).

Presented in part at the 43rd Annual Meeting of the American Society of Clinical Oncology, June 1–5, 2007, Chicago IL.

Study participants: The following institutions and investigators participated in the trial:

National Hospital Organization Dohoku Hospital (Yuka Fujita and Satoru Fujiuchi), Tochigi Cancer Center (Kiyoshi Mori and Yukari Kamiyama), National Cancer Center Hospital East (Kaoru Kubota, Yutaka Nishiwaki and Nagahiro Saijo), National Cancer Center Hospital (Noboru Yamamoto, Tomohide Tamura and Hideo Kunitoh), International Medical Center (Koichiro Kudo and Yuichiro Takeda), Cancer Institute Hospital (Takeshi Horai and Makoto Nishio), Kanagawa Cancer Center (Kazumasa Noda and Fumihiro Oshita), Yokohama Municipal Citizen’s Hospital (Koshiro Watanabe and Hiroaki Okamoto), Niigata Cancer Center Hospital (Akira Yokoyama and Yuko Tsukada), Gifu City Hospital (Yoshiyuki Sawa and Takashi Ishiguro), Aichi Cancer Center Hospital (Toyoaki Hida), National Hospital Organization Nagoya Medical Center (Hideo Saka), Kinki University Hospital (Kazuhiko Nakagawa and Isamu Okamoto) and Kyushu University Hospital (Yoichi Nakanishi and Koichi Takayama).
